# Economic Decisions for Others: An Exception to Loss Aversion Law

**DOI:** 10.1371/journal.pone.0085042

**Published:** 2014-01-13

**Authors:** Flavia Mengarelli, Laura Moretti, Valeria Faralla, Philippe Vindras, Angela Sirigu

**Affiliations:** Centre de Neuroscience Cognitive, CNRS, UMR 5229, Bron, France; University of Bologna, Italy

## Abstract

In everyday life, people often make decisions on behalf of others. The current study investigates whether risk preferences of decision-makers differ when the reference point is no longer their own money but somebody else money. Thirty four healthy participants performed three different monetary risky choices tasks by making decisions for oneself and for another unknown person. Results showed that loss aversion bias was significantly reduced when participants were choosing on behalf of another person compared to when choosing for themselves. The influence of emotions like regret on decision-making may explain these results. We discuss the importance of the sense of responsibility embodied in the emotion of regret in modulating economic decisions for self but not for others. Moreover, our findings are consistent with the Risk-as-feelings hypothesis, suggesting that self-other asymmetrical behavior is due to the extent the decision-maker is affected by the real and emotional consequences of his/her decision.

## Introduction

In a famous scene of the movie Wall Street: Money Never Sleeps (2010), Michael Douglas, interpreting the financial trader Gordon Gekko, defines moral hazard as “*when somebody takes your money and he is not responsible for it*”. Economists have proposed the notion of moral hazard to describe those situations in which “*one person makes the decision about how much risk to take, while someone else bears the costs if things go badly*” [1]. More generally the moral hazard indicates a situation involving two parties, one of whom is responsible for the interest of the other but has incentive to pursue his own [2].

Although most of the research on decision-making has especially focused on situations where subjects choose for themselves and therefore for their own interest [3], [4], in the real world people often delegate their decisions and make choices for others [5]. Principal-agent models have addressed this issue in the attempt to describe how risk taking is shared among parties in conditions where the principal engages the agent to take decisions in his/her own interest [6], [7]. This type of situations is quite common in the context of the western financial system where traders as Gordon Gekko represent a good example of decision-making on behalf of someone else. In other words, when we hand over our money to financial advisors, will they handle it as carefully as if it was their own money? Are individuals loss-averse when the money they make choice with is not their own money? It has been argued that people would assign a higher value on a good that they own compared to an identical good that they do not own [8], suggesting that loss of others' money would not trigger an equal amount of emotional arousal as loss of own money does.

Previous literature has already highlighted some potential self/other differences in decision-making under risk, even though findings are still controversial [9]. Some studies found more risk-averse behavior [10] or even no difference [11] in tasks involving decisions for others comparing to self. In contrast, others have showed that individuals are more incline to risk-seeking behavior when decisions involve other people than the self [12]–[14]. Of interest, in a recent report, Polman [15] investigated how loss aversion modulates choices for oneself and for others using different scenarios. He found significant diminished loss aversion when monetary decisions are referred to others comparing to those decisions referring to oneself.

Support for an asymmetry in self/other decisions comes also from neuroimaging studies that showed that while playing ultimatum game, activity in the medial prefrontal cortex (MPFC), a region implicated in socio-affective processes [16], was recruited selectively for unfair offers when these referred to self but not to others [17]. Consistently, previous literature has shown that affective processes strongly impact on our own decisions [18]–[21] and this may constitute the basis for the difference in self/other decisions. Indeed, a complex emotion like regret was found to affect human decision-making [22], [23]. It has been demonstrated that people tend to avoid risky decisions in order to anticipate the experience of regret [24], [25]. Specifically, the emotion of regret incorporates a process of counterfactual reasoning which refers to our ability to compare what happened with what could have happened but did not occur. In this context, regret arises when the obtained outcome of a decision is worse relative to the one obtained by the rejected alternative [25]. Importantly, regret embodies a sense of responsibility for the negative consequences of the agent's choice [26] and seems to increase for events in which the subject has active control on the choice and its consequences [27]–[29]. Here we hypothesize that this feeling of responsibility is suppressed or weakened in situations of moral hazard where people do not bear the consequences of their decisions, namely when they are choosing for others.

We tested participants in three economic choice tasks [30] in which decisions had to be made in two different conditions: “Self” - where subjects had to choose for themselves and their choice impacted only on their own final gain and “Other” - where subjects were delegated to choose on behalf of an unknown person and their choice impacted only on the final gain of this person. We suggest that when decisions have no direct consequences for the decision- maker, he/she would feel less responsible during the choice process and therefore less sensitive to the perspective of losing money. Following on this idea we expected choices made in the “Other” condition to be more prone to risk than those made in the “Self” condition.

## Materials and Methods

### Ethics Statements

This research was part of a large project on decision making approved by the local ethical committee Sud-Est IV, Léon Bérard (07027). The experiment was performed in accordance with the ethical standards laid down in the 1964 Declaration of Helsinki.

Thirty-four healthy volunteers (15 men; mean age  = 23.48 y, range: 19–30) participated in the study. All participants were students from Lyon University, and were recruited via an online recruitment system. Participants were not taking psychoactive medications, and were free of current or past psychiatric or neurological illnesses as determined by history. They were naïve as to the purpose of the study for which they gave their written consent.

The experiment was run in individual sessions. Participants performed three different lottery choice tasks. Each task was performed in two different conditions: (1) “Self” condition, where subjects had to choose for themselves and their choice impacted only on their own final gain; (2) “Other” condition, where subjects were delegated to choose on behalf of another unknown person and their choice impacted only on the final outcome of this person. Visual features and trial time course were similar across the two conditions with the exception of a picture (representing a fictive person) reminding the participant that he/she was performing in the “Other” condition. In the “Other” condition participants were told that they were choosing on behalf of someone else who was waiting in another room to remain anonymous. The order of the two conditions was counterbalanced across participants. Importantly, no information was provided to participants, either before or after the choice, on the other person's risk preferences.

Participants were informed they would be compensated for their participation proportionally to the amount of money gained during the “Self” condition. At the end of the experiment they all received a fixed amount of 20 Euros, irrespective of their performance in the tasks.

In task 1, they performed repeated binary choices between a sure option (loss or gain) and a gamble (loss or gain). There were six possible monetary outcomes in the gamble (50€, 100€, 200€, −50€, −100€, −200€), with a probability to occur varying from 1% to 99% within 13 possibilities. Both certain and uncertain options had the same expected value, as the certain amount corresponded always to the up-rounded value of the uncertain amount multiplied by the probability.

The six amount of money in the gamble (50€, 100€, 200€, −50€,−100€,−200€) appeared at least once for each possible probability, so that participants performed 78 trials in each condition.

In task 2, participants were asked to decide about the acceptability of a series of mixed prospects. They were instructed to indicate the minimum amount of money they were willing to accept as a sure gain to risk each of the 50% uncertain losses that were proposed. Twelve different amounts of loss were possible (−5€ −7€ −10€ −15€ −20€ −26€ −37€ −50€ −72€ −100€ −140€ −200€) forming an approximated geometric series from −5 to −200 € with a 1.414 ratio.

In task 3, subjects were asked to choose between two mixed gambles that both opposed a 50% chance gain to a 50% chance loss. In gamble 1, there were 4 possible couples of amounts. In gamble 2, the loss amount was approximately equal to 2.5 times the loss amount in gamble 1, while the gain amount varied from trial to trial. This task aimed to assess the increase of gain subjects felt necessary to compensate the increase of loss between the first and second gamble (30, 40, 60 and 75 €, see Table 1). The procedure involved two subsequent series of seven trials. In the first sequence, seven gains in gamble 2, forming a large-span geometrical progression, were presented to the subject in a pseudo-random order. The seven choices between gamble 1 and 2 were used for computing on-line a new geometrical series of seven gains with a smaller span than the first. This procedure was repeated 5 times for each of the 4 couples of gambles presented in a pseudo-random order during five subsequent blocks. Thus, the task consisted of a total of 280 trials (5×4×14) for each experimental condition. For each couple of gambles, the gain in gamble 2 the subject asked for compensating the increase of loss in gamble 2 with respect to gamble 1, was estimated by means of a logistic regression of its 70 individual responses (yes/no to gamble 2) with respect to the proposed gain in gamble 2.

**Table 1:** Problems for testing loss aversion.

Problem	Loss gamble1	Gain gamble 1	Loss gamble 2	Gain gamble 2
1	−20	50	−50	x
2	−30	80	−70	x
3	−40	110	−100	x
4	−50	150	−125	x

## Results

Since the across-subjects distribution of the quantitative data obtained in the three tasks (choice proportion and amounts) did not fit the usual Gaussian bell shapes (heavy tails), we systematically used non-parametric tests.

### Task 1

Wilcoxon signed-rank test was used to assess whether individual average proportion of risky choices were dependent on choice condition (“Self” *vs.* “Other”). Results revealed that subjects were significantly more biased to choose the risky option rather than the sure one in the “Other” (45%) relative to the “Self” condition (32%) (W = 91 p = 0.004).

**Figure 1:**
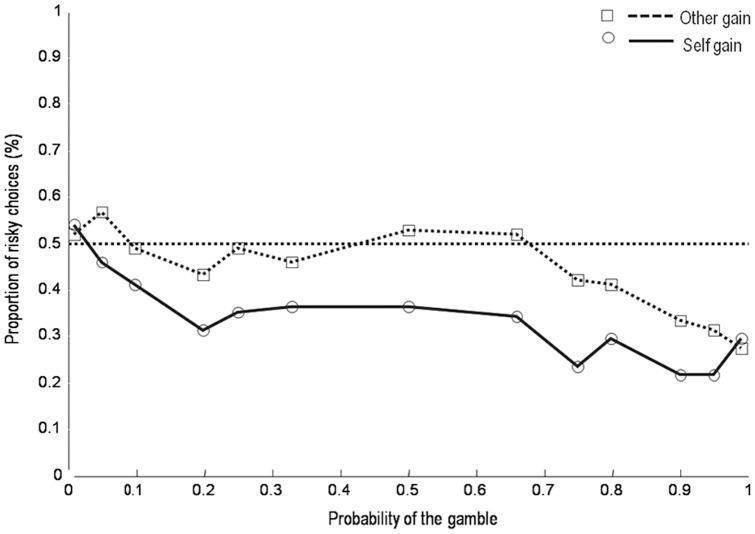
Proportion of participants' risky choices for each possible outcome probability in “Self” and “Other” conditions.

Following the procedure used by Tversky and Kahneman [30] we set 10% as the threshold between high and low probabilities. A Wilcoxon signed-rank test on the average proportion of risky choices for low (< = .10%) and high probabilities (> = .10%) of the risky gambles showed a significant difference in the proportion of risky choices between the “Self” and “Other” condition when the probability was high (W = 70, p = 0.002). No significant difference between the two conditions for the low probabilities (W = 176.5, p = 0.38) was found. The results from the first task demonstrate that subjects are significantly more risk-seeking when they chose on behalf of another person comparing to when they are choosing for themselves (see Figure 1).

### Task 2

**Figure 2:**
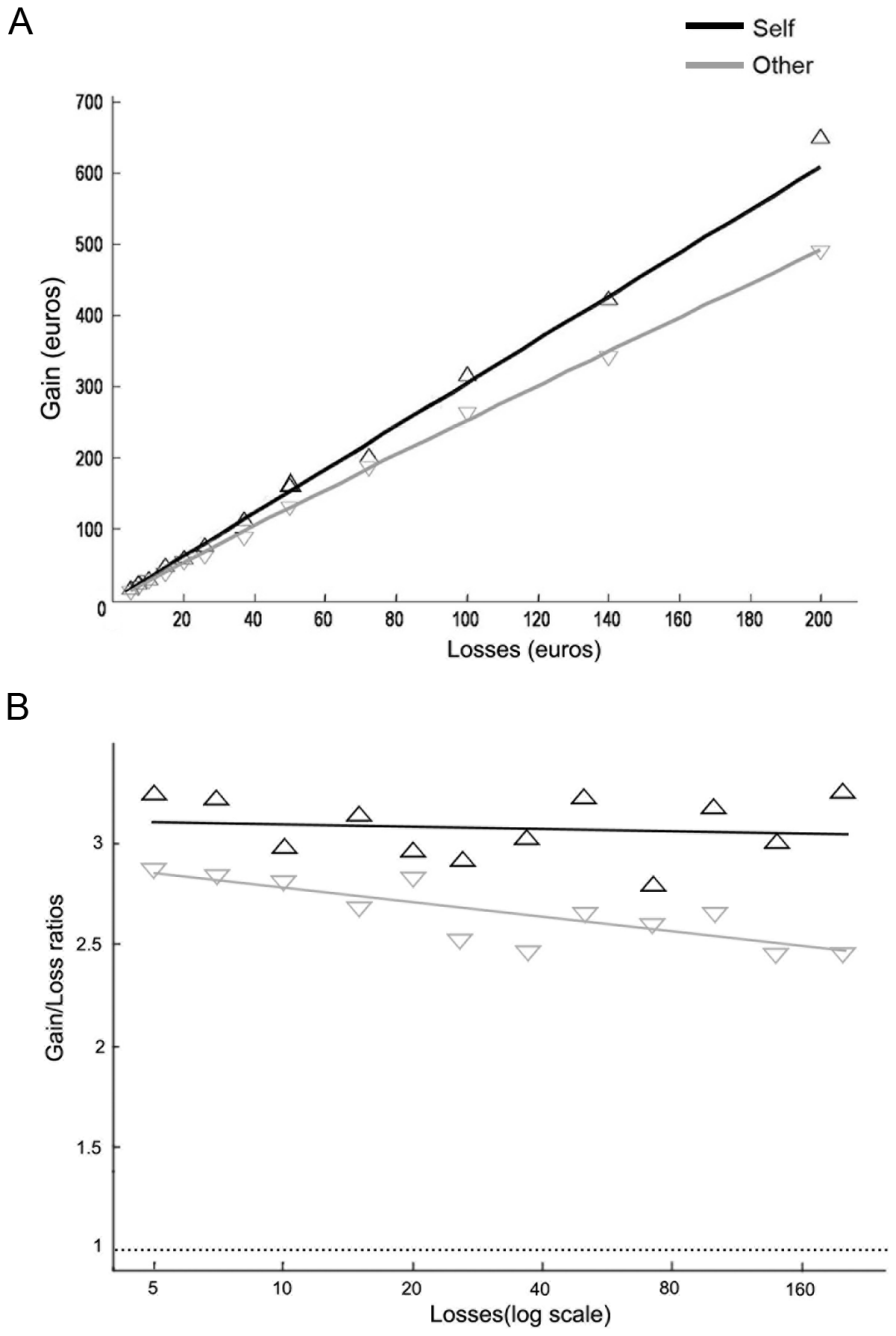
Panel A. Geometrical average of the gain participants were willing to accept in order to play the 50/50 gamble as a function of each of the twelve proposed losses, in the “Self” and “Other” conditions. Panel B. Geometric means of individual gain/loss ratios as a function of each of the twelve proposed losses (log scale) in the “Self” and “Other” conditions.

Figure 2- panel A illustrates the geometrical average of the gains participants were willing to accept in order to play the gamble as a function of the proposed losses, in both “Self” and “Other” conditions. We performed individual linear regressions of the logarithms of gain/loss ratio (hereafter “theta” from prospect theory [30]) with respect to the logarithm of the relative losses. The relative losses, defined as the losses divided by their geometrical average (31.16€), were used to obtain independent intercept and slope variables in the regressions. Thanks to this method, an intercept equal to zero would indicate that an individual asks a gain equal to the loss (log(1) = 0), and a slope equal to zero would mean that the gain/loss ratio does not (linearly) depend on the loss amount.

The intercepts of individual regressions showed that in the “Self” condition, participants on average required a gain amount equal to 3.07 times the loss amount (e.g. 614€ to expose to a 50% chance to lose 200€), whereas in the “Other” condition they were willing to accept the same gamble with 2.65 times the loss amount (e.g. 530€ for 200€). The difference between experimental conditions was highly significant (W  = 88, p  = 0.00061). In addition, the gain/loss ratio theta, considered as an index of loss aversion [(30)], tended to decrease with the loss amount in the “Other” but not in the “Self” condition (Figure 2 – panel B). Nevertheless, this was not confirmed by Wilcoxon test on individual slopes (Slope difference: W  = 178, p = 0.11065). The results from this task show that when facing the consequences of a potential loss, subjects requested higher gain for self and lower for others. It is worthwhile to note that, in line with empirical research on risk and inter-temporal preference [31], a gain-loss asymmetry was observed in both conditions so that the amount of gain that participants were willing to accept in order to play the gamble was two (Other) -three (Self) times higher than the prospected loss.

### Task 3

**Figure 3:**
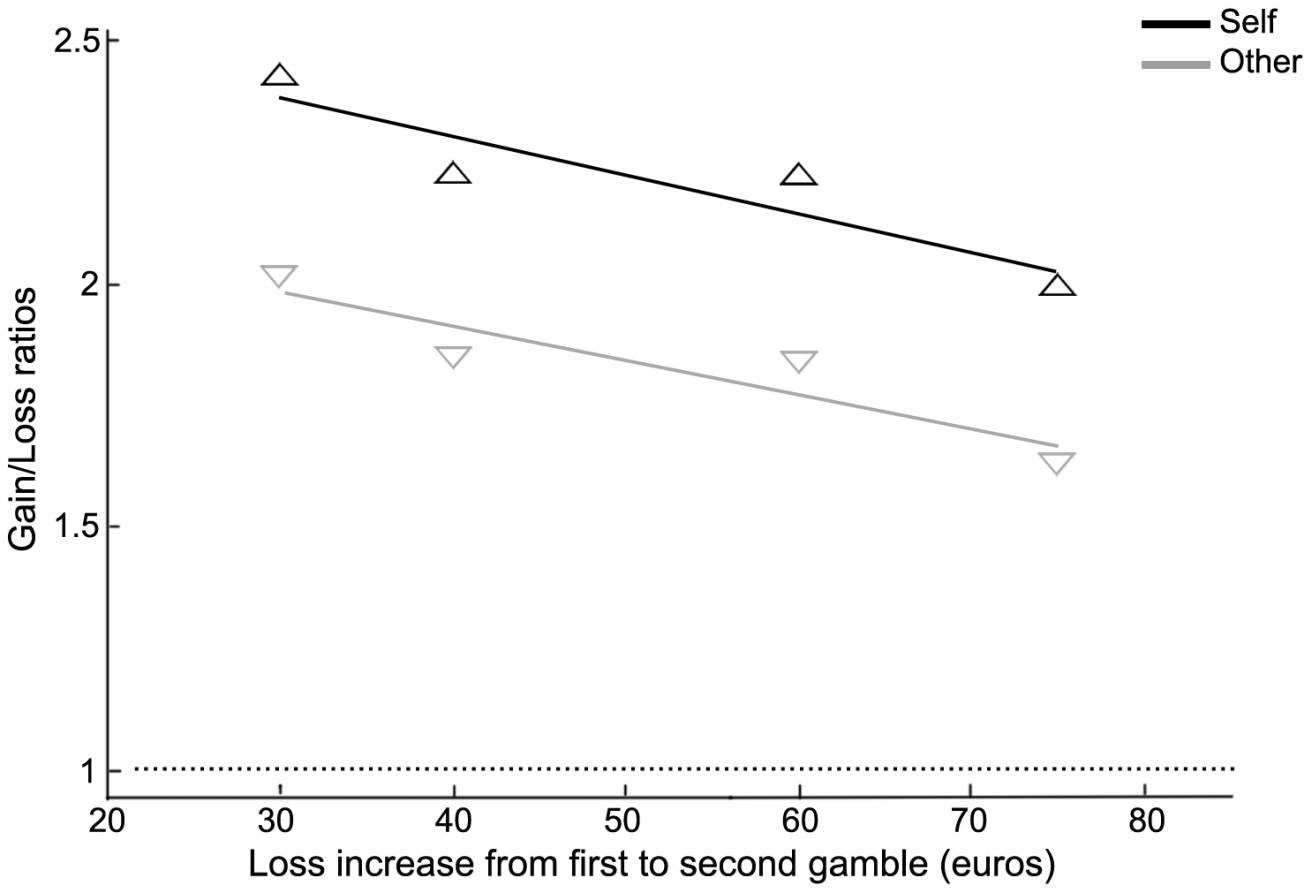
Geometric mean of individual loss aversions (gain/loss ratio) compensating for increasing loss differences between two gambles.

In the “Self” condition, individual logistic regressions showed that subjects preferred gamble 2 to gamble 1 when the gain in gamble 2 exceeded 127, 174, 253 and 312 € (across-individual geometrical average), which correspond to respective gain increases of 77, 94, 143 and 162 € with respect to the gains in gamble 1 (50, 80, 110 and 150€). In the “Other” condition, the requests of subjects were lower (second gamble gain: 113, 159, 230 and 286 €, respectively). Figure 3 represents the geometric mean of individual loss aversion, defined as (gain gamble2 - gain gamble1) / (loss gamble2 – loss gamble1). As in task 2, separately for “Self” and “Other” condition, we performed individual linear regressions of the logarithm of the loss aversion with respect to the log of relative losses in the first gamble (relative losses being defined as the increases in loss in the four couples divided by their geometrical mean).

The results showed that the geometric mean of individual loss aversion thetas were significantly higher for self (2.21) than for other (1.83) (W = 69, p = 0.00003) and did not vary as function of losses (Slope difference: W = 282.0 p = 0.80015).

## Discussion

In the current study three different financial decisions tasks were used to investigate the hypothesis that making economic choices for oneself and for another person involves distinct processes. We showed that when deciding on others' behalf, participants become more risk-seeking as compared to when deciding for themselves. This finding corroborates the hypothesis suggesting that economic decisions are perceived less riskier and loss aversion is minimized when economic consequences involve other people [12], [14], [15].

Situations in which people make decisions on behalf of other people under uncertainty are often associated with moral hazard problems. According to principal-agent theory [6], [32] in which the principal delegates the agent to work on his/her interest, economic efficiency is affected when the two parties have different risk preferences and asymmetric information (i.e. the agent has more information than the principal). Indeed, a loss of efficiency arises when the agent, whose action is not observable, deviates from principal's interests generating the so-called *agency costs*. This efficiency loss may be reduced by using suitable mechanisms aimed at lining up the interests of the two parties and reaching equilibrium between the principal and the agent, thus limiting the opportunistic behavior of the agent. In moral hazard models, where typically principal and agent are respectively risk neutral and risk averse, reducing agent's risk aversion may be possible by means of an incentive scheme enabling to achieve an equilibrium and assuring that the behavior of the agent conforms to principal's preference, i.e. risk neutral. The cost of the incentive will be higher the more the agent is risk averse. However, our paradigm presents some substantial differences from the situations addressed in a typical principal-agent problem. Indeed, our participants were completely blind relative to the other's risk preferences and did not receive any feedback or incentive, so that the choices of the decision-maker could not be influenced by the principal-agent contract.

Thus, why people do not make decisions for others as they would do for themselves?

The study by Polman [15] demonstrated that differences in loss aversion are influenced by psychological mechanisms (e.g. construal level, regulatory focus and/or information seeking) that also distinguish between self and other's choice, suggesting that cognitive biases are less incisive when people make decisions for others.

We go further by proposing the sense of responsibility incorporated in the process of decision-making as a possible explanation. The sense of responsibility in committing to an action has been previously connected to counterfactual thinking and regret [23], [25], [33] as well as the social emotion of guilt [34]–[36]. However, a recent study found higher level of loss aversion in self choices than in others, indicating that subjects were more motivated to avoid emotion like regret compared to guilt [37]. Possibly, regretting a bad choice for oneself has a much more intense emotional power than the feeling to regret a bad choice for another person. The activation of such mechanism may also fit with previous findings on group decision-making showing that when decisions are made considering other fellows in a group, risk is evaluated more rationally compared to individual decisions [38].

The discrepancy between self/other decision-making may also reflect different psychological processes, such affective and cognitive processes. Recent literature has provided theoretical and empirical evidence of a dual-process model of decision-making under risk and uncertainty [39]. This model proposes two separated ways to process information and to evaluate stimuli: one deliberative and rational, based on calculation and the other intuitive and automatic, based on feelings. It has been shown that in a wide range of situations people were insensitive to the magnitude of the stimulus when relying on their feelings but on the contrary they displayed relatively constant sensitivity to scope when relying on calculation. This explanation is also consistent with the Risk-as-feeling hypothesis [40] which claims that emotional reactions to risky situations often do not correspond to cognitive evaluations of those risks and when such a difference emerges, emotional reactions influence actual behavior more than cognition. Consistently, Civai and colleagues [41] reported that emotional ratings of fair and unfair offers were evaluated as stronger when deciding for the self than for a third-party and participants exhibited an increased emotional arousal when about to reject the unfair offers referring to themselves than when addressing a third-party. In addition, studies on the neural correlates of self/other decision-making found that activity of social affective areas such as medial prefrontal cortex (MPFC) are associated to self decision and not to decisions for another party [17]. In keeping with this, we suggest that self-other decisions are asymmetric, due to the extent real and emotional consequences affect the agent's decision. Unfortunately, our data do not provide any measure of emotional or cognitive bias so that this latter hypothesis remains at this time speculative, though it can be the core question of further investigations.

In summary, we provide empirical evidence in favor of the hypothesis that people do not choose for others as they would choose for themselves, at least in an economic context. As a result, our findings suggest that loss aversion as formalized by Kahneman and Tversky [30], [42] is not mandatory but rather dependent on the economic context. In light of these results, self/other asymmetry should be included among the number of variables (i.e. context, mood, feelings) that have already been demonstrated to affect risk preferences in decision-making. The discrepancy of self/other decision-making processes and loss aversion is an issue that applies to a vast spectrum of disciplines and domains of our life such as finance, law, management and even medicine. For this reason unveiling the psychological processes underlying this behavioral difference is of great importance for everyday life economic and social decisions.
